# Acute Coronary Syndromes and Nonalcoholic Fatty Liver Disease: “Un Affaire de Coeur”

**DOI:** 10.1155/2020/8825615

**Published:** 2020-11-30

**Authors:** Abdulrahman Ismaiel, Stefan-Lucian Popa, Dan L. Dumitrascu

**Affiliations:** 2nd Department of Internal Medicine, “Iuliu Hatieganu” University of Medicine and Pharmacy, Cluj-Napoca, Romania

## Abstract

**Background and Aims:**

Both nonalcoholic fatty liver disease (NAFLD) and ischemic heart disease have common pathogenic links. Evidence for the association of NAFLD with acute coronary syndromes (ACS), complex multivessel coronary artery disease (CAD), and increased mortality risk in ACS patients is still under investigation. Therefore, we conducted a systematic review aiming to clarify these gaps in evidence.

**Methods:**

We conducted a systematic search on PubMed and EMBASE with predefined keywords searching for observational studies published till August 2020. NAFLD diagnosis was accepted if confirmed through biopsy, imaging techniques, surrogate markers, or codes. Full articles that satisfied our inclusion and exclusion criteria were included in the systematic review. We used the NHLBI quality assessment tool to evaluate included studies.

**Results:**

Seventeen observational studies with a total study population of approximately 21 million subjects were included. Eleven studies evaluated whether NAFLD is an independent risk factor for developing ACS with conflicting results, of which eight studies demonstrated a significant association between NAFLD and ACS, mainly in Asian populations, while three reported a lack of an independent association. Conflicting results were reported in studies conducted in Europe and North America. Moreover, a total of five studies evaluated whether NAFLD and fatty liver severity in ACS patients are associated with a complex multivessel CAD disease, where all studies confirmed a significant association. Furthermore, seven out of eight studies evaluating NAFLD and hepatic steatosis severity as a predictor of all-cause and cardiovascular mortality and in-hospital major adverse cardiovascular events (MACE) in ACS patients demonstrated a significant independent association.

**Conclusions:**

NAFLD patients are associated with an independently increased risk of developing ACS, mainly in Asian populations, with inconsistent results in North American and European individuals. Moreover, NAFLD and hepatic steatosis severity were both independently correlated with complex multivessel CAD, mortality, and in-hospital MACE in ACS patients.

## 1. Introduction

Cardiovascular diseases (CVDs) account for about one-third of all deaths in the world, of which ischemic heart disease (IHD) is the greatest single cause of mortality worldwide, accounting for approximately 7 million deaths annually [1, 2]. Nonetheless, the prevalence of several metabolic disorders known to be risk factors for CVD such as nonalcoholic fatty liver disease (NAFLD), type 2 diabetes mellitus, dyslipidemia, and obesity has been rising dramatically lately [3, 4].

NAFLD is a multisystem complex pathology without current approved therapies, primarily affecting the liver which causes modifications to the structure and function of the liver, leading to an increased liver-related morbidity and mortality from cirrhosis, liver failure, and hepatocellular carcinoma [5–7]. Moreover, an increasing body of evidence supports that NAFLD is not only a progressive liver disease, but can also lead to multiple systemic consequences and extrahepatic manifestations, including effects exerted on the cardiovascular system (CVS) [8–11].

Interestingly, despite being a liver pathology, most deaths among NAFLD patients are due to CVD, mainly attributed to ischemic heart disease [12, 13]. Current evidence points out that NAFLD should be considered a significant independent risk factor for clinical and subclinical CVD, increased CVD-related morbidity, and all‐cause mortality [11–13]. Furthermore, the probability that NAFLD may be not only a marker but also an early mediator of atherosclerosis has been lately discussed [14]. However, several studies reported that NAFLD per se may not be causally leading to an increased cardiovascular (CV) risk [15–18].

Acute coronary syndrome (ACS) is a term that refers to any group of clinical symptoms consistent with acute myocardial ischemia. This includes unstable angina (UA), non-ST segment elevation myocardial infarction (NSTEMI), and ST-segment elevation myocardial infarction (STEMI) [19]. ACS and sudden death cause most IHD-related deaths representing 1.8 million deaths per year. The risk of acute coronary events in life is linked with the exposure to traditional cardiovascular risk factors [19]. These risk factors have also been demonstrated to also increase the susceptibility of the rapidly growing pathology, NAFLD [20–22].

Lately, several studies evaluated whether NAFLD is a predictor for an increasing risk of developing ACS, complexity of coronary artery disease (CAD), and increased mortality risk in ACS patients. However, results have been unclear with inconsistent results. Accordingly, we conducted the first systematic review to the best of our knowledge evaluating the association, complexity of CAD, all-cause and CV mortality risk, major adverse cardiovascular events (MACE), and adverse CV events of ACS in NAFLD patients through performing a systematic review.

## 2. Methods

This systematic review and meta-analysis was written according to the Preferred Reporting Items for Systematic Reviews and Meta-Analyses (PRISMA) guidelines [23].

### 2.1. Data Sources and Search Strategy

To identify potentially eligible observational studies evaluating ACS in NAFLD patients, we conducted a systematic search of PubMed and Embase from inception till the 4th of August 2020 without restrictions. The search strategy applied in these two databases included the following search string for PubMed ((“Acute Coronary Syndrome”[Mesh]) OR (“acute coronary syndrome”) OR (“Myocardial Infarction”[Mesh]) OR (“myocardial infarction”) OR (“ST Elevation Myocardial Infarction”[Mesh]) OR (“ST elevation myocardial infarction”) OR (“STEMI”) OR (“Non-ST Elevated Myocardial Infarction”[Mesh]) OR (“non-ST elevated myocardial infarction”) OR (“NSTEMI”)) AND ((“Non-alcoholic Fatty Liver Disease”[Mesh]) OR (“nonalcoholic fatty liver disease”) OR (“NAFLD”) OR (“NASH”) OR (“MAFLD”) OR (“Metabolic associated fatty liver disease”) OR (“Metabolic-dysfunction-associated fatty liver disease”)) and the following search string for Embase (‘acute coronary syndrome'/exp OR ‘acute coronary syndrome' OR ‘myocardial infarction'/exp OR ‘myocardial infarction' OR ‘st elevation myocardial infarction'/exp OR ‘st elevation myocardial infarction' OR ‘stemi' OR ‘non-st elevated myocardial infarction'/exp OR ‘non-st elevated myocardial infarction' OR ‘nstemi') AND (‘non-alcoholic fatty liver disease'/exp OR ‘nonalcoholic fatty liver disease' OR ‘nafld' OR ‘nash' OR ‘mafld' OR ‘metabolic associated fatty liver disease' OR ‘metabolic-dysfunction-associated fatty liver disease'). Moreover, in order to minimize results bias, we manually searched the reference lists of pertinent articles in order to identify any additional relevant missed publications.

### 2.2. Study Selection and Eligibility Criteria

All observational studies evaluating the association, complexity of coronary artery disease, MACE, and mortality risk of ACS in NAFLD patients were eligible for inclusion. Original articles were included in the qualitative assessment and systematic review if they met the following inclusion criteria: (1) observational cohort population-based/hospital-based/primary care-based, case-control, descriptive studies of prospective or retrospective design; (2) hepatic steatosis confirmed based on one of the following methods: biopsy, imaging techniques such as ultrasonography, computed tomography (CT), magnetic resonance imaging (MRI), surrogate or noninvasive biomarkers of NAFLD, liver enzymes, or codes such as International Classification of Diseases (ICD); (3) confirmed diagnosis of ACS according to each study definition; (4) adult subjects (aged ≥18 years) without restrictions in terms of gender, race, or ethnicity; and (5) studies conducted on humans only.

Exclusion criteria included the following: (1) significant alcohol consumption or the presence of other secondary causes of hepatic steatosis; (2) patients with confirmed hepatitis virus of any etiology; (3) other known causes of CLD; (4) patients with confirmed cirrhosis of any etiology; (5) subjects with end-stage liver disease who are awaiting or underwent liver transplantation; (6) studies published in languages other than English, German, and Romanian; and (7) case reports, reviews, practice guidelines, commentaries, opinions, letters, editorials, short surveys, articles in press, conference abstracts, conference papers, and abstracts published without a full article.

According to the abovementioned eligibility criteria, two investigators (A.I. and S.L.P.) performed a screening evaluation independently through scrutinizing titles and abstracts excluding any apparently irrelevant studies. Subsequently, selected articles fulfilling the inclusion and exclusion criteria were further evaluated by carefully reviewing the full text. A mutual consensus was reached by discussion to resolve any discrepancies regarding study eligibility.

### 2.3. Data Extraction

We extracted the following information from eligible studies: author's name, publication year, study location, study population, the source of cohort, sample size, mean age, ACS prevalence, the approach to diagnose hepatic steatosis, the number of NAFLD cases, gender, body mass index (BMI), and aspartate aminotransferase (AST) and alanine aminotransferase (ALT) levels, in addition to the follow-up duration and main study findings. One investigator (A.I.) extracted the data through an electronic spreadsheet, and then another investigator (S.L.P.) reviewed the extracted data for accuracy. Discrepancies regarding the results of extracted data were settled by discussion. Extracted data was then entered into tables, while final data was collated and presented in the text of the manuscript.

### 2.4. Quality Assessment

Two investigators (A.I. and S.L.P.) used the National Heart, Lung, and Blood Institute (NHLBI) to independently perform the quality assessment for included studies in order to assess bias risk and internal validity in individual studies in a similar manner [24]. One tool was used for observational cohort and cross-sectional studies. The evaluation assessment tool items were answered by “yes”, “no”, “not applicable”, “cannot determine”, or “not reported”. Subsequently, the studies received a rating as “good”, “fair”, or “poor” upon completion of the evaluation. Any discrepancies regarding quality assessment evaluation results of the two investigators were handled by discussion. Eligibility of the studies was not affected by the results of methodological quality assessment.

## 3. Results

### 3.1. Literature Search

**Figure 1:**
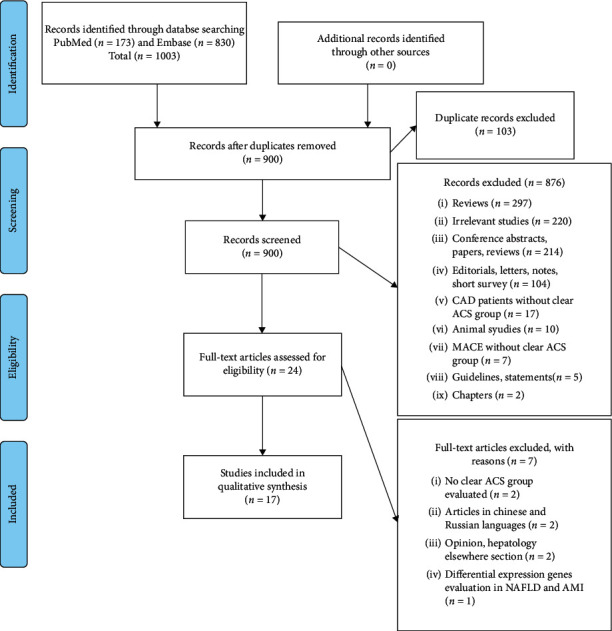
PRISMA flow diagram for search and selection processes of this systematic review.

The literature search identified 173 and 830 records from PubMed and Embase, respectively. Following the removal of 103 duplicates, we obtained a total of 900 records that were carefully reviewed through the assessment of the titles and abstracts, of which a total of 876 records were excluded due to the following reasons: (1) two hundred and ninety-seven review articles; (2) two hundred and twenty irrelevant articles; (3) two hundred and fourteen conference abstracts, papers, or reviews; (4) one hundred and four editorials, letters, notes, and short surveys; (5) seventeen articles describing CAD without clear ACS; (6) ten studies conducted on animals; (7) seven articles evaluating major adverse cardiovascular events (MACE) without clear ACS; (8) five guidelines and statements; and (9) two chapters. The eligibility of the remaining 24 articles according to the inclusion and exclusion criteria was evaluated through assessing the full text, of which seven records were excluded due to the following: (1) no clear ACS group in NAFLD patients [25, 26]; (2) opinion [27]; (3) manuscripts in Chinese and Russian languages [28, 29]; (4) article evaluating the differential expression genes of NAFLD and in acute myocardial infarction datasets [30]; and (5) an article published under hepatology elsewhere section where the full article is already included in our systematic review [31]. Hence, a total of 14 records fulfilled our inclusion and exclusion criteria and were included in our qualitative assessment and systematic review as described in Figure 1 [32–48].

### 3.2. Study Characteristics

The main characteristics of included studies are summarized in Table 1. A total of approximately 21 million subjects were included in this review. The number of NAFLD cases varied from 54 to 120,795, while the ACS cases varied between 80 and 16,574 with a follow-up period ranging from 6 months to 17 years in the included studies.

Six studies had a cohort study design (retrospective cohort study [35, 46], prospective cohort study [36, 37], prospective population-based cohort study [41], matched cohort study [42], nationwide population-based cohort study [43], and cohort study [44, 47]). Moreover, two studies had a cross-sectional study design (cross-sectional study [32] and cross-sectional analysis of a prospective single-center study [45]) and two observational studies (retrospective observational study [34, 40]). Furthermore, we also included a descriptive study [39] and three studies that did not clearly specify their study design [32, 38, 48].

Eight studies were conducted in Europe (Turkey *n* = 4, Italy *n* = 1, Germany *n* = 1, Finland *n* = 1, and multiple countries *n* = 1), five studies in Asia (Republic of Korea *n* = 2, China *n* = 1, Armenia *n* = 1, and Sri Lanka *n* = 1), and four studies in North America (USA *n* = 2 and Canada *n* = 2).

### 3.3. Quality Assessment

We used the NHLBI quality assessment tools to evaluate the methodological quality of eligible studies included in the qualitative assessment and systematic review as demonstrated in Table 2. Seven studies had an overall rating of “good” [33, 34, 36, 41–44], eight studies were rated “fair” [32, 35, 37, 39, 40, 45, 46, 48], and two studies were rated “poor” [38, 47]. Generally, all included studies clearly stated a research question or objective. The study population was specified and defined as who, where, and when in thirteen studies [33–35, 37, 39–46, 48] while six studies had a sufficient time frame [34, 41–44, 46]. Moreover, only one study evaluated hepatic steatosis more than once over the study period partially for a group of participants [34]. All but five studies assessed potential cofounding variables and adjusted statistically for their impact [37, 38, 45–47]. Furthermore, some included studies did not report a few items evaluated in the quality assessment tools.

Four out of the seven studies rated as “good” evaluating the relationship between NAFLD and ACS demonstrated a significant association between NAFLD and ACS [33, 36, 43, 44] while three studies reported a lack of an independent association [34, 41, 42]. The remaining four studies supporting this relationship were rated as “fair” [45, 46] and “poor” [38, 47]. Moreover, the association between NAFLD and complexity of CAD in ACS patients was evaluated in five studies, out of which only one was rated as “good” [33], three as “fair” [32, 45, 48], and one as “poor” [38], all supporting a more severe CAD in ACS patients with NAFLD. Furthermore, the relationship between NAFLD and adverse CV events, in-hospital MACE, all-cause mortality, and CV mortality in ACS patients was evaluated in eight studies. Two out of the three of the studies rated as “good” supported this association [36, 43] and one study opposed it [34]. The remaining five studies that supported this association were rated as “fair” [35, 37, 39, 40, 48].

### 3.4. Definition of NAFLD

Hepatic steatosis was evaluated using ultrasonography for diagnosing NAFLD in most studies (*n* = 10) [32, 33, 36–40, 45, 46, 48], while the others studies used codes (*n* = 3) [42, 44, 47], fatty liver index (FLI) (*n* = 2) [41, 43], elevated ALT levels (*n* = 1) [35], and non-contrast CT imaging (*n* = 1) [34].

### 3.5. NAFLD as a Predictor for Developing ACS

Several studies evaluated whether NAFLD is an independent risk factor for developing ACS with conflicting results. A total of eleven studies evaluated this association, where eight studies demonstrated a significant association between NAFLD and ACS while three reported a lack of an independent association.  Table 3 summarizes the current available data evaluating the association between ACS and NAFLD.

Boddi et al. evaluated 95 consecutive nondiabetic patients admitted to cardiac intensive care unit for STEMI demonstrating a very high prevalence of NAFLD evaluated using ultrasonography in the studied group [33]. A prospective cohort study conducted by Emre et al. on 186 nondiabetic patients undergoing PCI for STEMI [36]. They concluded that in-hospital nonfatal myocardial infraction (MI) was significantly greater in patients with an FLD ≥3 score (*p*=0.011). Furthermore, Ozturk et al. compared patients with MI, stable CAD, and normal coronary arteries reporting that MI occurred predominantly in NAFLD patients evaluated using ultrasonography compared to patients with stable CAD [38]. Moreover, Kim et al. conducted a Korean nationwide population-based cohort study on 3,011,588 subjects demonstrating a HR for nonfatal MI of 2.16 (95% CI: 2.01–2.31) comparing the lowest to the highest FLI quartiles with similar results after performing a stratified analysis by age, sex, use of dyslipidemia medication, obesity, diabetes, and hypertension [43]. They concluded that FLI, a surrogate marker for NAFLD, is an independent predictor for developing acute MI. A cohort study conducted on a primary care population by Labenz et al. on 44,096 individuals demonstrated that MI patients had a significantly higher frequency of NAFLD compared to controls (2.9% vs. 2.3%, *p* <  0.001) with an obtained HR of 1.34 (*p*=0.003) for incidence of MI in all NAFLD patients on regression analysis concluding that NAFLD is an independent risk factor for MI in primary care in Germany [44]. A cross-sectional analysis of a prospective single-center study conducted by Montemezzo et al. on 139 ACS patients concluded that NAFLD is common in ACS patients, compromising about 60% of their study population [45]. Furthermore, a retrospective cohort study conducted by Sinn et al. conducted on 111,492 individuals using a Korean healthcare database of adults over 40 years of age without any significant history of CVD, liver disease, or cancer at baseline with a total of 725,706.9 person-years of follow-up demonstrated that the cumulative incidence of MI was consistently higher in participants with NAFLD evaluated using ultrasonography compared to controls during the whole follow-up period after adjusting for established CV risk factors and medications [46]. A cohort study involving 13,290 patients with NAFLD conducted by Vandromme et al. concluded that NAFLD subtype 2 was associated with MI with an HR of 6.6 (95% CI: 3.3–13.3, *p* <  0.001) [47].

On the other hand, Dunn et al. conducted a retrospective observational study involving 2,343 type 2 diabetic patients reporting that a history of baseline myocardial infarction patients was significantly more frequent in patients with <30% hepatic steatosis evaluated using non-contrast CT imaging [34]. Moreover, a prospective population-based cohort study by Olubamwo et al. involving 1,205 STEMI patients demonstrated that incident acute MI was associated with a high FLI category with an HR of 1.65 (95% CI: 1.22–2.23) in the minimally adjusted model [41]. However, more comprehensive models including metabolic factors demonstrated a nonsignificant HR of 1.136 (95% CI: 0.777–1.662) suggesting that the predictability of acute MI using FLI might be due to several metabolic factor interactions. Furthermore, a matched cohort conducted in Netherlands, Spain, and UK by Alexander et al. involving 17.7 million individuals demonstrated a pooled HR for acute MI of 1.17 (95% CI: 1.05–1.30) after adjusting for age and smoking in NAFLD or NASH patients compared to controls [42]. Nonetheless, in a group of subjects with more details on risk factors, the HR for acute MI was 1.01 (95% CI: 0.91–1.12) after adjusting for established cardiovascular risk factors concluding that NAFLD is not independently associated with acute MI.

### 3.6. Complexity of CAD in ACS Patients with NAFLD

A total of five studies evaluated whether the presence of NAFLD is associated with a more complex CAD disease in ACS patients, where all studies demonstrated a more severe CAD assessed using SYNTAX, GRACE, and Gensini scores and angiography in NAFLD patients.

A cross-sectional study conducted by Agac et al. involving 80 ACS patients demonstrated that NAFLD patients presented with a significantly higher SYNTAX score (18 ± 8 vs. 11 ± 5, *p* value = 0.001). Moreover, the ultrasonographic stage of NAFLD was significantly correlated with SYNTAX score by univariate analysis (*r* = 0.6, *p* <  0.001), while the presence of NAFLD was found to be an independent factor associated with supramedian SYNTAX score with an OR of 13.20 (95% CI: 2.52–69.15) concluding that NAFLD patients present with a more complex CAD [32]. Moreover, Boddi et al. demonstrated that nondiabetic STEMI patients with severe fatty liver disease were younger in age and presented with an increased prevalence of multivessel CAD compared to patients with mild NAFLD assessed by ultrasonography (*P* <  0.01), while severe fatty liver disease was independently associated with an increased threefold risk of multivessel CAD by logistic regression analysis [33]. A study conducted by Ozturk et al. involving 224 patients demonstrated that patients with MI had an increased frequency of NAFLD with stable CAD, in addition to a significant association between hepatic steatosis severity evaluated by ultrasonography with the severity of CAD assessed using Gensini score (*r* = 0.648, *p* <  0.001) [38]. A cross-sectional analysis of a prospective single-center study conducted by Montemezzo et al. concluded that NAFLD severity detected by ultrasonography is strongly related to the complexity of CAD on angiography [45]. Furthermore, Xia et al. conducted a study involving 325 acute MI patients over 60 years of age where they concluded that NAFLD is related to the severity of CAD in elderly subjects with acute MI [48].

### 3.7. MACE in ACS Patients with NAFLD

A total of eight studies evaluated MACE in ACS patients with NAFLD, out of which, seven reported that NAFLD is a predictor of all-cause and CV mortality and in-hospital MACE in ACS patients, while one study opposed this association.

A retrospective cohort study conducted by Ravichandran et al. involving 528 ACS patients with a follow-up period of 6 months demonstrated that NAFLD determined using elevated serum ALT is associated with an increased risk of adverse outcomes and all-cause mortality up to 6 months after discharge with an adjusted OR of 8.96 (95% CI: 3.28–24.49) in ACS patients [35]. Moreover, Emre et al. concluded that in-hospital nonfatal MI and death were both significantly increased in patients presenting a FLD ≥3 score (*p*=0.011 and 0.041, resp.). They also conducted a multivariate analysis where an FLD ≥3 score was found to be independent predictor of in-hospital MACE with an OR of 2.454 (95% CI: 1.072–4.872, *p*=0.048) [36]. Furthermore, Kocharyan et al. conducted a prospective cohort on 166 STEMI and NSTEMI patients with a 12-month follow-up period demonstrating that NAFLD is associated with an increased mortality (*p* < 0.01) in acute MI patients, while there was no association between the presence of NAFLD and rehospitalizations (*p* > 0.05) [37]. Perera et al. conducted a study on 120 nonfatal ACS patients concluding that NAFLD patients presented with an increased predicted mortality during in-ward stay with an adjusted OR of 31.3 (95% CI: 2.2–439.8, *p*=0.011) and after 6 months from discharge with an adjusted OR of 15.59 (95% CI 1.6–130.6, *p*=0.011) recommending a more aggressive treatment of CAD in NAFLD patients [39]. In addition, Keskin et al. conducted a retrospective observational study involving 360 STEMI patients reporting an in-hospital mortality rates for grade 0, 1, 2, and 3 NAFLD evaluated using ultrasonography of 4.7%, 8.3%, 11.3%, and 33.9%, respectively [40]. After a follow-up of three years, mortality rates for grade 0, 1, 2, and 3 NAFLD were 5.6%, 7.8%, 9.5%, and 33.3%, respectively. Moreover, in-hospital mortality risks were higher in grade 3 NAFLD patients using a multivariable hierarchical logistic regression analysis with an OR of 4.2 and an HR of 4.0 in a multivariable Cox proportional regression analysis. Kim et al. concluded in a nationwide population-based cohort study that FLI is an independent predictor of CV mortality with an HR of 1.98 (95% CI: 1.9–2.06). The results remained similar even after performing stratified analyses of established cardiovascular risk factors [43]. Moreover, Xia et al. reported that acute MI patients with NAFLD had a lower ejection fraction and higher rates of adverse cardiovascular event [48].

On the other hand, Dunn et al. reported that hepatic steatosis lacks the predictive value for nonfatal adverse cardiovascular outcomes in a study population involving type 2 diabetic patients [34].

## 4. Discussion

Recently, there is a rapidly growing interest in determining whether NAFLD and its severity are associated with ACS. To the best of our current knowledge, this is the first systematic review to evaluate the association, complexity of CAD, all-cause and CV mortality risk, in-hospital MACE, and adverse CV events of ACS in NAFLD patients. Our systematic review included 17 studies with a total study population of approximately 21 million individuals reporting results associating NAFLD with an increased independent risk for developing ACS in Asian populations. However, this independent association was inconsistent in European and North American individuals after adjusting for established CV risk factors. Moreover, we also reported a significant association relating a more advanced FLI with acute MI. Furthermore, NAFLD and hepatic steatosis severity were both significantly correlated with a more complex CAD, increased mortality, and in-hospital MACE in ACS patients. Most of these findings were demonstrated to be independently associated with NAFLD regardless of the established traditional CV risk factors across a wide range of patient populations.

In our systematic review, we reported several findings that need to be further discussed. Firstly, in order to reflect our current knowledge about NAFLD, this term was recently updated to metabolic-dysfunction-associated fatty liver disease (MAFLD) with newly defined diagnostic criteria [49, 50]. However, these two terms, NAFLD and MAFLD, should not be used interchangeably due to the existing differences between them. All studies evaluated in the current systematic review used the diagnostic criteria of NAFLD and not MAFLD; therefore, our findings reflect the association in NAFLD and not MAFLD. Interestingly, MAFLD definition was demonstrated to be more practical for identifying fatty liver disease (FLD) patients with an increased risk of disease progression [51].

Secondly, we observed a variety of methods that were used to detect hepatic steatosis and diagnose NAFLD. A positive diagnosis of NAFLD can be confirmed through confirming the presence of hepatic steatosis by histology which is the current gold standard, as well as imaging methods such as ultrasonography which is the most common imagistic assessment used, CT scans and MRI, in addition to noninvasive assessment through surrogate markers [20, 52]. Most studies included in our systematic review used ultrasonography for diagnosing NAFLD. Despite demonstrating a low sensitivity when hepatic steatosis is less than 20% on biopsy, ultrasonography remains the preferred initial first-line imaging method for assessing liver fat with a sensitivity and specificity of 84.8% and 93.6%, respectively [53, 54]. Moreover, a couple of studies used surrogate markers to evaluate hepatic steatosis including FLI and ALT levels. The FLI was demonstrated to be a simple and accurate predictor of hepatic steatosis in the general population [55]. On the other hand, evidence demonstrated that solo use of liver enzymes such as ALT levels is a poor predictor of NAFLD as approximately 70–80% of patients may have normal range levels and therefore is not helpful for diagnosing or evaluating the severity of the disease [56, 57].

Thirdly, we noticed that most included studies supported the presence of an independent association linking NAFLD with an increased risk of ACS. However, three studies opposed this association, out of which one study was a matched cohort study involving 17.7 million European individuals demonstrating the presence of this association which lost its significance after adjusting for established CV risk factors in a group of subjects with more complete data on risk factors. Although studies conducted on European and American populations reported inconsistent results, interestingly, all studies conducted on Asian populations reported a significant independent association between NAFLD and an increased risk of ACS. This might be explained by the different lifestyles and epidemiological characteristics as well as eating habits compared with Western subjects. Therefore, taking into consideration the different populations with distinct key contributing characteristics should not be neglected while elaborating the current results. Another explanation that might be attributing to these inconsistent results can be explained by the common mutual CV risk factors such as obesity, diabetes, dyslipidemia, genes, and other parameters that are present in both diseases.

Fourthly, the complexity of CAD in ACS was assessed using several different methods including thorough angiography, in addition to the SYNTAX, GRACE, and Gensini scores. All these methods have been demonstrated to be useful in evaluating the severity and extent of atherosclerosis in CAD patients presenting with ACS [58, 59].

Fifthly, an independent relationship linking increased in-hospital MACE and all-cause and CV mortality in ACS patients with NAFLD and hepatic steatosis severity was reported in most studies. However, only one study opposed this association which was conducted on type 2 diabetic patients [34]. Therefore, the results obtained in this study cannot be generalized on the general population.

Sixthly, the quality assessment of studies included in our systematic review demonstrated that the majority of studies that are currently published in the literature evaluating the association of interest are of “fair” quality making up eight studies out of seventeen, followed by seven studies that were rated as “good” and only two studies rated as “fair”. Therefore, results obtained by studies with “fair” and “poor” ratings should be interpreted with caution because of the increased risk of bias and possible methodological flaws.

Our systematic review has several limitations which should be mentioned. First, the observational design of the studies included in this review does not allow us to establish a clear causal correlation between NAFLD and ACS, complexity of CAD, or mortalities. Second, most included studies assessed hepatic steatosis using ultrasonography and to a lesser extent FLI, ALT levels, and CT, whereas none of the studies used liver biopsy which is the current gold standard for diagnosing and staging of NAFLD. This can possibly under- or overestimate the prevalence of NAFLD. However, we did not exclude studies using surrogate markers or liver enzymes as we wanted our study to be thorough and comprehensive by covering all studies published till the search date evaluating the studied associations. Hence, we can have more generalizable results with more significance. Third, despite having two included studies of “poor” quality, most included studies were rated as either “fair” or “good”, therefore associating the results with a lower risk of bias.

Nevertheless, our systematic review also presents several important strengths. The topic of this systematic review is of important clinical relevance due to the rapid increase of prevalence in NAFLD worldwide, in addition to the higher related morbidity and mortality associated with ACS. We believe that the current review outlines and summarizes the current literature. It also points out the missing required data to be evaluated in further future studies. Moreover, this systematic review was conducted comprehensively, therefore, covering the current published studies evaluating the studied associations in a systematic manner. To the best of our knowledge, this is the first systematic review to evaluate the association, complexity of CAD, and all-cause and CV mortality in ACS patients with NAFLD.

## 5. Conclusions and Future Directions

In conclusion, NAFLD patients are associated with an independently increased risk of developing ACS, mainly in Asian populations. However, this association was inconsistent in studies conducted on individuals from North American and European backgrounds. Moreover, NAFLD and hepatic steatosis severity were both demonstrated to be independently correlated with complex multivessel CAD, all-cause and CV mortality, in addition to in-hospital MACE in ACS patients.

Therefore, due to the higher predicted MACE and mortality rates in ACS patients with FLD, we recommend screening for hepatic steatosis using the newly defined MAFLD diagnostic criteria in order to identify FLD patients with an increased risk for disease progression, also requiring a thorough CV risk assessment. Early monitoring and identification of patients with MAFLD will allow enhancing the management plans and modifying the underlying risk factors, reducing the overall incidence of adverse events and improving the overall prognosis as well as promoting survival. Furthermore, FLD patients from different racial backgrounds should be evaluated accordingly while stratifying for CV risk, especially in ACS, due to the different contributing distinct characteristics that should not be neglected.

## Figures and Tables

**Table 1:** Studies assessing the outcomes associated with NAFLD in patients with ACS.

First author/year/country	Study design	Study characteristics	Main findings
Agac et al./2013/Turkey [32]	Cross-sectional study	(i) Total subjects: 80	NAFLD patients presented a significantly higher SYNTAX. Moreover, the stage of NAFLD correlated with SYNTAX score. In multivariate binary logistic analysis, the presence of NAFLD was an independent factor associated with supramedian SYNTAX score. In conclusion, NAFLD is a predictor of a more complex CAD in ACS patients.
		(ii) Population: ACS patients	
		(iii) ACS prevalence: STEMI: 29 (36.3%); NSTEMI: 41 (50.6%); unstable angina: 10 (12.5%)	
		(iv) NAFLD: 65 (81.25%)	
		(v) Mean age (years): 62.2 ± 11.2	
		(vi) Gender (males): 75 (78.9%)	
		(vii) BMI: NAFLD: 28.6 ± 2.1; NAFLD absent: 25.1 ± 1.8	
		(viii) NAFLD diagnosis: ultrasonography	
		(ix) AST level: —	
		(x) ALT level: NAFLD 35 ± 17; NAFLD absent 19 ± 7	
		(xi) SYNTAX score: NAFLD 18 ± 8; NAFLD absent 11 ± 5	
		(xii) Follow up: —	

Boddi et al./2013/Italy [33]	Unclear	(i) Total subjects: 95	Compared to nondiabetic STEMI patients with mild FLD, severe FLD patients were younger in age and presented a higher prevalence of multivessel CAD at logistic regression analysis; severe FLD was independently associated with a threefold risk of multivessel CAD.
		(ii) Population: nondiabetic STEMI patients	
		(iii) ACS prevalence: STEMI: 95 (100%)	
		(iv) NAFLD: 83 (87.36%)	
		(v) Mean age (years): 62.2 ± 11.2	
		(vi) Gender (males): 75 (78.9%)	
		(vii) BMI: All patients: 26.0 ± 2.6; score <3 : 25.0 ± 2.5; score ≥3 : 27.2 ± 2.3	
		(viii) NAFLD diagnosis: ultrasonography	
		(ix) AST level: all patients: 80 (48–183); score <3 : 76 (50–200); score ≥3 : 80 (38–183)	
		(x) ALT level: all patients: 45 (30–68); score <3 : 32 (24–100); score ≥3 : 53 (38–68)	
		(xi) Follow-up: —	

Dunn et al./2013/USA [34]	Retrospective observational study	(i) Total subjects: 2,343	Hepatic steatosis was not associated with any nonfatal adverse CV outcomes.
		(ii) Population: type 2 diabetic patients	
		(iii) ACS prevalence: MI overall: 653 (28%); <30% steatosis: 599 (28%); ≥30% steatosis: 54 (233%)	
		(iv) NAFLD: 78 (3.33%) using ICD-9 codes; <30% steatosis: 2110; ≥30% steatosis: 233	
		(v) Mean age (years): <30% steatosis: 66.6 ± 15.1; ≥30% steatosis: 58.1 ± 13.7	
		(vi) Gender (males): 1,078 (46%)	
		(vii) BMI: <30% steatosis: 30.8 ± 7.5; ≥30% steatosis: 36.7 ± 8.5	
		(viii) NAFLD diagnosis: non–contrast CT imaging	
		(ix) AST level: <30% steatosis: 22 (17, 34); ≥30% steatosis: 26 (18, 39)	
		(x) ALT level: —	
		(xi) Follow-up: 5 years	

Ravichandran et al./2014/Canada [35]	Retrospective cohort study	(i) Total subjects: 528	NAFLD is determined by increased ALT levels, is associated with in-hospital all-cause mortality, and up to 6 months after discharge in ACS patients.
		(ii) Population: ACS patients	
		(iii) ACS prevalence: STEMI: 288 (49.3%); NSTEMI 191 (31.7%); unstable angina 76 (13%); other 29 (5%)	
		(iv) NAFLD: 54 (10.23%)	
		(v) Mean age (years): 63.4 (12.4)	
		(vi) Gender (males): 402 (74.6%)	
		(vii) BMI: —	
		(viii) NAFLD diagnosis: elevated ALT level >90th percentile	
		(ix) AST level: —	
		(x) ALT level: multivariable linear regression was used to determine the change in maximum measured cardiac troponin I (cTnI) per each 1 IU/l increase in serum ALT concentration.	
		(xi) Follow-up: 6 months	

Emre et al./2015/Turkey [36]	Prospective cohort study	(i) Total subjects: 186	In-hospital nonfatal MI and death were significantly higher in patients with an FLD score ≥3. Using multivariate analysis, FLD score ≥3 was an independent predictor of in-hospital MACE.
		(ii) Population: nondiabetic patients who underwent PCI for STEMI	
		(iii) ACS prevalence: STEMI: 186 (100%)	
		(iv) NAFLD: FLD score <3 : 111 (59.68%); FLD score ≥3 : 75 (40.32%)	
		(v) Mean age (years): 58 ± 11	
		(vi) Gender (males): 142 (76%)	
		(vii) BMI: all patients: 26.5 ± 2.4; score <3 : 26.0 ± 2.4; score ≥3 : 27.3 ± 2.2	
		(viii) NAFLD diagnosis: ultrasonography	
		(ix) AST level: all patients: 79 ± 35; score <3 : 76 ± 35; score ≥3 : 82 ± 35	
		(x) ALT level: all patients: 45 ± 20; score <3 : 42 ± 19; score ≥3 : 48 ± 20	
		(xi) Follow-up: **—**	

Kocharyan/2016/Armenia [37]	Prospective cohort study	(i) Total subjects: 166	The presence of NAFLD in acute MI patients is associated with increased mortality.
		(ii) Population: STEMI and NSTEMI patients	
		(iii) ACS prevalence: STEMI and NSTEMI: 166 (100%)	
		(iv) NAFLD: 91 (54.82%)	
		(v) Mean age (years): 63 ± 0.96	
		(vi) Gender (males): 116 (69.88%)	
		(vii) BMI: —	
		(viii) NAFLD diagnosis: ultrasonography	
		(ix) AST level: —	
		(x) ALT level: —	
		(xi) Follow-up: 12 months	

Ozturk et al./2016/Turkey [38]	Unclear	(i) Total subjects: 224	NAFLD was more prevalent in MI patients compared to stable CAD patients. Moreover, NAFLD was also significantly associated with CAD severity. Significant correlations between Gensini score and hepatic steatosis grade were reported.
		(ii) Population: group 1: patients with an MI-STEMI and NSTEMI; group 2: patients with stable CAD; and group 3: patients with normal coronary artery	
		(iii) ACS prevalence: group 1: 94 (100%); STEMI: 70 (74.5%); and NSTEMI: 24 (25.5%)	
		(iv) NAFLD: overall: 101 (45%); group 1: 66 (70.2%); group 2: 23 (38.3 %); and group 3: 12 (17.1 %)	
		(v) Mean age (years): group 1: 60.3 ± 13.2; group 2: 57.1 ± 9.5; and group 3: 55.9 ± 7.4	
		(vi) Gender (males): 160 (71.43%)	
		(vii) BMI: group 1: 25.5 ± 3.2; group 2: 25.2 ± 2.5; and group 3: 24.6 ± 3.3	
		(viii) NAFLD diagnosis: ultrasonography	
		(ix) AST level: —	
		(x) ALT level: —	
		(xi) Gensini score: group 1: 118 ± 23; group 2: 51 ± 17; and group 3: 0	
		(xii) Follow-up: **—**	

Perera et al./2016/Sri Lanka [39]	Descriptive study	(i) Total subjects: 120	Patients with NAFLD have a higher predicted mortality from ACS during in-ward stay and at 6 months after discharge.
		(ii) Population: nonfatal ACS	
		(iii) ACS prevalence: STEMI-NAFLD: 16 (28.6); NAFLD absent: 16 (25.0); total: 32 (26.7); *p*=0.659	
		NSTEMI-NAFLD: 40 (71.4); NAFLD absent: 48 (75.0); total: 88 (73.3)	
		(iv) NAFLD: 56 (46.67%)	
		(v) Mean age (years): 61.28 ± 11.83	
		(vi) Gender (males): 75 (62.5%)	
		(vii) BMI: 24.64 ± 9.8	
		(viii) NAFLD diagnosis: ultrasonography	
		(ix) AST level: —	
		(x) ALT level: NAFLD: 62.9 ± 46.2; NAFLD absent: 29.4 ± 11.9; total: 44.9 ± 36.5	
		(xi) GRACE score: NAFLD: 120.2 ± 26.9; NAFLD absent: 92.3 ± 24.2; *p* < 0.001	
		(xii) Follow-up: 6 months	

Keskin et al./2017/Turkey [40]	Retrospective observational study	(i) Total subjects: 360	In STEMI patients, the presence of NAFLD is correlated with unfavorable clinical outcomes, out of which, grade 3 NAFLD patients were found to have the highest mortality rates.
		(ii) Population: STEMI patients	
		(iii) ACS prevalence: STEMI: 360 (100%)	
		(iv) NAFLD: 191 (53.06%)	
		(v) Mean age (years): 59 ± 12	
		(vi) Gender (males): 241 (66.94%)	
		(vii) BMI: NAFLD absent: 27.1 ± 3.4; grade 1 NAFLD: 26.7 ± 3.4; grade 2 NAFLD: 27.0 ± 3.8; grade 3 NAFLD: 27.8 ± 3.6	
		(viii) NAFLD diagnosis: ultrasonography	
		(ix) AST level: absent NAFLD: 30 ± 17; grade 1 NAFLD: 33 ± 25; grade 2 NAFLD: 33 ± 25; and grade 3 NAFLD: 36 ± 22	
		(x) ALT level: absent NAFLD: 24 ± 21; grade 1 NAFLD: 30 ± 24; grade 2 NAFLD: 31 ± 21; and grade 3 NAFLD: 36 ± 26	
		(xi) SYNTAX score: absent NAFLD: 7 ± 2; grade 1 NAFLD: 14 ± 5; grade 2 NAFLD: 20 ± 9; and grade 3 NAFLD: 26 ± 9	
		(xii) Follow-up: 3 years	

Olubamwo et al./2018/Finland [41]	Prospective population-based cohort study	(i) Total subjects: 1,205	Incident CVD can be predicted using FLI. However, predicting acute MI using FLI was not demonstrated to be an independent association, mainly due to several metabolic factor interactions.
		(ii) Population: STEMI patients	
		(iii) ACS prevalence: acute MI: 269 (22.32%)	
		(iv) NAFLD: 648 (53.78%)	
		(v) Mean age (years): FLI <30: 51.5 (5.8); FLI 30 to <60: 52.7 (5.7); and FLI ≥60: 51.49 (5.8)	
		(vi) Gender (males): 1,205 (100%)	
		(vii) BMI: FLI <30: 24.3 (1.9); FLI 30 to <60: 27.3 (1.9); and FLI ≥60: 30.9 (3.3)	
		(viii) NAFLD diagnosis: FLI	
		(ix) AST level: —	
		(x) ALT level: —	
		(xi) Follow-up: 17 years	

Alexander et al./2019/Italy, Netherlands, Spain, and UK [42]	Matched cohort study	(i) Total subjects: 17.7 million	NAFLD does not appear to be associated with acute MI risk after adjustment for established cardiovascular risk factors.
		(ii) Population: population-based, electronic primary healthcare database	
		(iii) ACS prevalence: Acute MI-NAFLD: 1,035; controls: 67,823	
		(iv) NAFLD: 120,795 (0.7%)	
		(v) Mean age (years): Italy—NAFLD: 55.6 (14.2); controls: 54.6 (13.5); Netherlands—NAFLD: 56.1 (13.6); controls: 55.6 (13.3); Spain—NAFLD: 55.6 (13.3); controls: 54.2 (12.9); and UK—NAFLD: 53.3 (13.1); controls: 52.9 (13.2)	
		(vi) Gender (males): Italy—NAFLD: 57.2%; controls: 54.9%; Netherlands—NAFLD: 48.6%; controls: 48.1%; Spain—NAFLD: 52.5%; controls: 48.8%; and UK—NAFLD: 51.1%; controls: 50.4%	
		(vii) BMI: Italy—NAFLD: 29.7 (5.0); controls: 27.5 (5.0); Netherlands—NAFLD: 31.0 (5.4); controls: 28.3 (5.2); Spain—NAFLD: 31.4 (5.1); controls: 28.7 (5.1); and UK—NAFLD: 32.4 (5.9); controls: 28.5 (5.9)	
		(viii) NAFLD diagnosis: ICD-9 codes, codes for HSD, ICPC Dutch for IPCI, ICD-19 and Read codes	
		(ix) AST level: Italy—NAFLD: 24 (19–33); controls: 20.7 (17–25); Netherlands—NAFLD: 29 (22–40); controls: 23 (20–28); Spain—NAFLD: 29 (22–40); controls: 21 (18–27); and UK—NAFLD: 32 (24–47); controls: 22 (19–27)	
		(x) ALT level: Italy—NAFLD: 30 (20–49); controls: 21 (16–30); Netherlands—NAFLD: 37 (25–56); controls: 25 (18–33); Spain—NAFLD: 35 (23–54); controls: 20 (15–28); and UK—NAFLD: 46 (29–69); controls: 23 (17–31)	
		(xi) Follow-up: 2.1–5.5 years	

Kim et al./2020/Republic of Korea [43]	Nationwide population-based cohort study	(i) Total subjects: 3,011,588	FLI is an independent predictor for developing MI and CV mortality.
		(ii) Population: nationwide population-based	
		(iii) ACS prevalence: Acute MI: 16,574 (0.55%)	
		(iv) NAFLD: According to FLI quartiles	
		(v) Mean age (years): 51.86 ± 8.20	
		(Vi) Gender (males): 1,290,580 (42.9%)	
		(vii) BMI: 23.82 ± 2.91	
		(viii) NAFLD diagnosis: FLI	
		(ix) AST level: —	
		(x) ALT level: —	
		(xi) Follow-up: median of 6 years	

Labenz et al./2020/Germany [44]	Cohort study	(i) Total subjects: 44,096	NAFLD constitutes an independent risk factor for MI in primary care in Germany.
		(ii) Population: primary care population	
		(iii) ACS prevalence: acute MI-NAFLD: 2.9%; controls: 2.3%; *p* < 0.001	
		(iv) NAFLD: 22,048 (50%)	
		(v) Mean age (years): 55.6 (13.4)	
		(vi) Gender (males): 50.2%	
		(vii) BMI: **—**	
		(viii) NAFLD diagnosis: ICD-10 codes	
		(ix) AST level: —	
		(x) ALT level: —	
		(xi) Follow-up: 10 years	

Montemezzo et al./2020/Canada [45]	Cross-sectional analysis of a prospective single-center study	(i) Total subjects: 139	NAFLD is common in ACS patients. The ultrasonographic severity of NAFLD is strongly associated with the complexity of coronary artery obstruction evaluated on angiography.
		(ii) Population: ACS patients	
		(iii) ACS prevalence: STEMI: 40 (59.7%); NSTEMI: 51 (36.6%); and UA 48 (34.3%)	
		(iv) NAFLD: 76 (55.2%)	
		(v) Mean age (years): overall: 59.7; CAD: 59 ± 11.62; without CAD: 54.3 ± 10.83	
		(vi) Gender (males): 83 (59.7%)	
		(vii) BMI: **—**	
		(viii) NAFLD diagnosis: ultrasonography	
		(ix) AST level: with CAD: 75.6 ± 116.46; without CAD: 35.6 ± 28.42	
		(x) ALT level: with CAD: 55.4 ± 44.13; without CAD: 105.3 ± 147.12	
		(xi) Follow-up: —	

Sinn et al./2020/Korea [46]	Retrospective cohort study	(i) Total subjects: 111,492	NAFLD was associated with a higher incidence of MI independently of established risk factors. Moreover, this finding was similar in patients in the presence and absence of more advanced NAFLD evaluated by NFS.
		(ii) Population: healthcare database of adults over 40 years old without history of CVD, liver disease, or cancer at baseline	
		(iii) ACS prevalence: MI: 183 (with an overall incidence of 2.5 cases per 10,000 person-years	
		(iii) NAFLD: 37,263 (33.42%)	
		(iv) Mean age (years): 52.0 (8.1)	
		(v) Gender (males): 57,123 (51.2%)	
		(vi) BMI: 23.7 (2.9)	
		(vii) NAFLD diagnosis: ultrasonography	
		(viii) AST level: —	
		(ix) ALT level: —	
		(x) Follow-up: 725,706.9 person-years of follow-up	

Vandromme et al./2020/USA [47]	Cohort study	(i) Total subjects: 13,290	NAFLD subtype 2 was correlated with MI. When considering subtype 1 as the reference, subtype 5 was independently linked to the highest risks for MI compared to all other subtypes. Moreover, subtype 2 was also independently related to an increased risk of MI.
		(ii) Population: hospital database of NAFLD patients using electronic signatures of disease	
		(iii) ACS prevalence: —	
		(iv) NAFLD: 13,290 (100%)	
		(v) Mean age (years): 53 ± 14.7	
		(vi) Gender (males): 49.4%	
		(vii) BMI: **—**	
		(viii) NAFLD diagnosis: ICD-9, ICD-10, current procedural terminology, and medication mapping	
		(ix) AST level: —	
		(x) ALT level: —	
		(xi) Follow-up: —	

Xia et al./2020/China [48]	Unclear	(i) Total subjects: 325	NAFLD is associated with the severity of CAD, as well as being an independent predictor of adverse CV events in elderly patients with acute MI.
		(ii) Population: acute MI patients over the age of 60 years	
		(iii) ACS prevalence: 100%	
		(iv) NAFLD: 111 (34.15%)	
		(v) Mean age (years): 70.24 ± 9.46	
		(vi) Gender (males): 182 (56%)	
		(vii) BMI: **—**	
		(viii) NAFLD diagnosis: ultrasonography	
		(ix) AST level: —	
		(x) ALT level: —	
		(xi) Follow-up: —	

ACS: acute coronary syndrome; ALT: alanine aminotransferase; CAD: coronary artery disease; CT: computer tomography; CV: cardiovascular; CVD: cardiovascular disease; FLD: fatty liver disease; FLI: Fatty Liver Index; ICD: International Classification of Diseases; ICPC: International Classification of Primary Care; MI: myocardial infarction; NAFLD: nonalcoholic fatty liver disease; NFS: NAFLD Fibrosis Score; NSTEMI: non-ST-segment elevation myocardial infarction; PCI: percutaneous coronary intervention; and STEMI: ST-segment elevation myocardial infarction.

**Table 2:** NHLBI quality assessment tool for observational cohort and cross-sectional studies.

Criteria	Agac et al. [32]	Boddi et al. [33]	Dunn et al. [34]	Ravichandran et al. [35]	Emre et al. [36]	Kocharyan [37]	Ozturk et al. [38]	Perera et al. [39]	Keskin et al. [40]	Olubunmi et al. [41]	Alexander et al. [42]	Kim et al. [43]	Labenz et al. [44]	Montemezzo et al. [45]	Sinn et al. [46]	Vandromme et al. [47]	Xia et al. [48]
*(1) Was the research question or objective in this paper clearly stated?*	Yes	Yes	Yes	Yes	Yes	Yes	Yes	Yes	Yes	Yes	Yes	Yes	Yes	Yes	Yes	Yes	Yes

*(2) Was the study population clearly specified and defined?*	No	Yes	Yes	Yes	No	Yes	No	Yes	Yes	Yes	Yes	Yes	Yes	Yes	Yes	No	Yes

*(3) Was the participation rate of eligible persons at least 50%?*	NR	Yes	Yes	NR	Yes	NR	NR	Yes	NR	Yes	Yes	Yes	Yes	NR	Yes	NR	NR

*(4) Were all the subjects selected or recruited from the same or similar populations (including the same time period)? Were inclusion and exclusion criteria for being in the study prespecified and applied uniformly to all participants?*	CD	Yes	Yes	Yes	Yes	Yes	NR	Yes	Yes	Yes	No	Yes	Yes	Yes	Yes	Yes	Yes

*(5) Was a sample size justification, power description, or variance and effect estimates provided?*	No	Yes	No	No	No	No	No	No	No	No	No	No	No	Yes	No	No	Yes

*(6) For the analyses in this paper, were the exposure(s) of interest measured prior to the outcome(s) being measured?*	No	No	No	No	No	No	No	No	No	Yes	Yes	Yes	Yes	No	Yes	No	No

*(7) Was the time frame sufficient so that one could reasonably expect to see an association between exposure and outcome if it existed?*	No	No	Yes	No	No	No	No	No	No	Yes	Yes	Yes	Yes	No	Yes	No	No

*(8) For exposures that can vary in amount or level, did the study examine different levels of the exposure as related to the outcome (e.g., categories of exposure, or exposure measured as continuous variable)?*	Yes	Yes	Yes	Yes	Yes	No	Yes	No	Yes	Yes	No	Yes	No	Yes	No	No	No

*(9) Were the exposure measures (independent variables) clearly defined, valid, reliable, and implemented consistently across all study participants?*	Yes	Yes	Yes	Yes	Yes	Yes	Yes	Yes	Yes	Yes	Yes	Yes	Yes	Yes	Yes	Yes	Yes

*(10) Was the exposure(s) assessed more than once over time?*	No	No	Partially (81 subjects)	No	No	No	No	No	No	No	No	No	No	No	No	No	No

*(11) Were the outcome measures (dependent variables) clearly defined, valid, reliable, and implemented consistently across all study participants?*	Yes	Yes	Yes	Yes	Yes	Yes	Yes	Yes	Yes	Yes	Yes	Yes	Yes	Yes	Yes	Yes	Yes

*(12) Were the outcome assessors blinded to the exposure status of participants?*	Yes	Yes	NR	CD	NR	NR	Yes	NR	NR	NR	NA	NA	NA	Yes	NA	NA	NR

*(13) Was loss to follow-up after baseline 20% or less?*	NA	NA	NA	NA	Yes	Yes	NA	NA	NA	Yes	NA	NA	NA	NA	NA	NA	NA

*(14) Were key potential confounding variables measured and adjusted statistically for their impact on the relationship between exposure(s) and outcome(s)?*	Yes	Yes	Yes	Yes	Yes	No	No	Yes	Yes	Yes	Yes	Yes	Yes	No	No	No	Yes

*Rating*	Fair	Good	Good	Fair	Good	Fair	Poor	Fair	Fair	Good	Good	Good	Good	Fair	Fair	Poor	Fair

**Table 3:** Evidence evaluating the association between ACS and NAFLD.

Condition	Country	Study population	Evidence of association	Observation
Acute myocardial infarction	USA [34]	2,343	Lack of association	Demonstrating a lack of significant association in type 2 diabetic patients only.
	Netherlands, Spain, and UK [42]	17.7 million	Weak	Significant association after adjustment for age and smoking. However, the significance was lost after adjusting for systolic blood pressure, type 2 diabetes, total cholesterol level, statin use, and hypertension.
	Turkey [38]	224	Strong	NAFLD was more frequent in MI patients.
	Korea [43]	3,011,588	Strong	FLI significantly associated with MI even after performing stratified analyses by body weight, cholesterol, age, sex, use of dyslipidemia medication, obesity, diabetes, and hypertension.
	Germany [44]	44,096	Strong	Significant association even after performing regression analysis.
	Korea [46]	111,492	Strong	Significant association even after performing adjustments for age, sex, year of visit, smoking status, alcohol intake, BMI, systolic blood pressure, fasting glucose, LDL cholesterol, use of antihypertensive medications, use of antidiabetic medications, use of lipid-lowering medications, and use of aspirin and antithrombotic medications at baseline.
	USA [47]	13,290	Strong	NAFLD subtypes 2 and 5 were independently significantly associated with MI.

STEMI	Finland [41]	1,205	Weak	FLI is associated with MI in minimally adjusted models. However, it lost significance in most comprehensive models with metabolic factors.
	Italy [33]	95	Strong	High prevalence of NAFLD in nondiabetic patients admitted for STEMI.
	Turkey [36]	186	Strong	Severe FLD is an independent predictor of STEMI by performing multivariate analysis.

ACS	Canada [45]	139	Strong	60.5% of severe CAD patients had NAFLD.

ACS: acute coronary syndrome; FLD: fatty liver disease; FLI: Fatty Liver Index; LDL: low-density lipoproteins; MI: myocardial infarction; NAFLD: nonalcoholic fatty liver disease; and STEMI: ST-segment elevation myocardial infarction.
